# Relationship between postpartum depression and lactation status at a Japanese perinatal center: A cross-sectional study

**DOI:** 10.12688/f1000research.20704.2

**Published:** 2020-01-30

**Authors:** Shunji Suzuki

**Affiliations:** 1 Department of Obstetrics and Gynecology, Japanese Red Cross Katsushika Maternity Hospital, Tateishi, Katsushika-ku, Tokyo, 124-0012, Japan

**Keywords:** postpartum depression, lactation status, exclusive breastfeeding, the Edinburgh Postnatal Depression Scale, Japan

## Abstract

**Background:  **Some studies have demonstrated that breastfeeding can protect mothers from postpartum depression; therefore, we examined the association between postpartum depression and lactation status at one month after delivery at a Japanese perinatal center.

**Methods:** We reviewed the obstetric records of all (total 809) nulliparous healthy women with vaginal singleton delivery at 37-41 weeks’ gestation at our institute between July 2018 and June 2019. A face-to-face interview with the women was conducted on admission for delivery to ask whether or not they hoped to perform exclusive breastfeeding for their babies, and an additional interview was conducted one month after delivery to ask about their feeding methods currently. Maternal mental status was examined based on the scores using the Edinburgh Postnatal Depression Scale (EPDS), and women with EPDS scores of ≥9 points were regarded as ‘positive screening’.

**Results: **592 women (73.1%) hoped to perform exclusive breastfeeding for their babies on admission. Of these, at one month, 442 (74.7%) performed exclusive breastfeeding, while 150 (25.3%) performed mixed or artificial feeding. The average EPDS scores and the incidence of EPDS scores ≥9 points in the women performing exclusive breastfeeding were 4.3 ± 3.6 and 14.3% (63/442), respectively. They did not differ from those in the women performing mixed or artificial breast feeding [4.2 ± 3.7, p = 0.60 and 13.3% (20/150), p = 0.78].

**Conclusion:** Development of postpartum depression does not seem to be associated with incomplete breastfeeding at our hospital, and therefore there are other risk factors indicated in the development of postpartum depression.

## Introduction

Exclusive breastfeeding for the first 6 months of life has been recommended because of important health, medical, social, and developmental benefits to both mothers and babies ^1^. Postpartum depression has been recognized as the leading medical complication among new mothers ^2, 3^. To date, some risk factors for postpartum depression, such as personal and family factors, socioeconomic status, support from other family members and personal plans for furthering careers, have been examined ^4– 8^. Some studies have demonstrated that breastfeeding can protect mothers from postpartum depression and are starting to clarify which biological and psychological processes may explain this protection ^9– 11^. In addition, a short duration of breastfeeding has been reported to be associated with the development of postpartum depression ^11^.

In Japan, the breastfeeding rate at Japan’s baby-friendly hospitals (BFHs) at one month of age has been reported to be more than 75% ^12^; however, inconsistent knowledge of breastfeeding benefits and inappropriate hospital practices has been reported to be associated with the increased use of infant formula and reduced breastfeeding duration, although the national breastfeeding rates had been higher than other countries of similar health status ^13^. Unfortunately, only 50% of women who delivered at Japanese Red Cross Katsushika Maternity Hospital, a non-BFH institute, have performed exclusive breastfeeding for their babies at one month after delivery ^14, 15^. Recently, in Japan, the population of elderly and/or high-risk pregnant women has been increased, and the rate of exclusive breastfeeding may be expected to decrease ^4^. To examine the necessity of breastfeeding promotion in relation to maternal mental status, we examined the association between lactation status and postpartum depression at one month after delivery in Japanese women.

## Methods

The protocol for this analysis was approved by the Ethics Committee of the Japanese Red Cross Katsushika Maternity Hospital (approval number, K2018001). Written informed consent was obtained from each woman to participate in this study at the first meeting, i.e. before birth.

### Participants

We reviewed the obstetric records of all nulliparous healthy women (n = 809) with vaginal singleton delivery at 37–41 weeks’ gestation at Japanese Red Cross Katsushika Maternity Hospital between July 2018 and June 2019.

To control confounding factors, we excluded cases of multiparous women, multiple births, cesarean deliveries, mothers with a habit of smoking and/or drinking, mothers with pregnancy depression, mothers without partners mothers whose babies are low birth weight, and mothers whose babies were admitted to the neonatal intensive care unit (NICU) because they have been already reported to be associated with the prevalence of exclusive breastfeeding and/or postpartum depression ^4, 11, 16– 18^.

### Data collection

A face-to-face interview was conducted with the women on admission for delivery to ask them whether or not they hoped to perform exclusive breastfeeding for their babies at the delivery room of the hospital, and an additional interview was conducted one month after delivery to ask about their feeding methods at that time at the outpatient examination room of the hospital during routine check-up appointments.

Maternal mental status was examined, at one month after delivery, based on the scores of the questionnaires of the Edinburgh Postnatal Depression Scale (EPDS) at the same time of the interview. In this study, women with the EPDS scores of ≥9 points were regarded as ‘positive screening = 50% possibility of depression’, according to the results of previous observations in Japan by Okano *et al.* ^19, 20^.

### Data analysis

Data are presented as the mean ± SD or number (%). SPSS Statistics software version 20 (IBM Csorp., Armonk, NY, USA) was used for statistical analyses. For statistical analysis, the *Χ ^2^* test for categorical variables and Student’s *t*-test for continuous variables were used. Differences with *p* < 0.05 were considered significant.

## Results

On admission, 592 women (73.1%) hoped to perform exclusive breastfeeding for their babies and who met the conditions to be considered in the current study. Of these, 442 (74.7%) performed exclusive breastfeeding at one month, while 150 (25.3%) performed mixed or artificial feeding (mixed feeding: 296, artificial feeding: 24). There were no significant differences in maternal age between the two groups (exclusive breastfeeding: 32.4 ± 6.1 years; mixed or artificially feeding: 32.8 ± 6.4 years; *p* = 0.11).

The average EPDS scores and the incidence of EPDS scores of ≥9 points in the women performing exclusive breastfeeding were 4.3 ± 3.6 and 14.3% (63/442), respectively. These did not differ from those in the women performing mixed or artificial feeding [4.2 ± 3.7, *p* = 0.60 and 13.3% (20/150), *p* = 0.78]. In addition, the average EPDS score and the incidence of EPDS scores of ≥9 points in the women performing exclusive artificial feeding was 4.0 ± 3.2 and 8.3% (2/24), respectively.

## Discussion

The current results seemed to be contrary to those in some previous studies indicating that breastfeeding can protect mothers from postpartum depression ^9– 11^. In this study, a trend of higher score of EPDS was observed in the women with exclusive breastfeeding group, though the results were non-significant. The current results seemed to be unpredictable for us.

To date, lower plasma oxytocin levels leading to incomplete breastfeeding have been reported to be associated with the development of postpartum depression. In a recent study by Lara-Cinisomo *et al.* ^10^, for example, lower levels of plasma oxytocin were observed in women who had stopped breastfeeding and had postpartum depression by two months postpartum. The influence of synthetic oxytocin on a new mother’s well-being has been also reported previously ^10, 21, 22^. Oxytocin is released across the breastfeeding cycle, and oxytocin release has observed to exhibit a temporary anxiolytic-like calming effect on postpartum maternal mood disturbances ^21^. Therefore, oxytocin is believed to mediate a calming effect on postpartum mood in primiparous mothers with breastfeeding.

However, in this study, exclusive breastfeeding did not contribute to the prevention of postpartum depression significantly. The mental status of mothers considered to have low levels of oxytocin associate with incomplete breastfeeding seemed to be stable. Therefore, in social environments and/or clinical characteristics of pregnant Japanese women, there may be some serious risk factors for postpartum depression other than the status of breastfeeding, such as personal and family factors, socioeconomic status, support from other family members and personal plans for furthering careers ^4– 8^. For example, in our recent Japanese study that asked mothers’ biggest worry at two weeks after delivery, only 10% reported anxiety about breastfeeding ^8^. Although we believe that Japan is not a poor country, recently there are some morbid pregnant women who have been reduced to poverty ^23^.

We understand the small sample of the current study is a serious limitations. A study in women who gave birth at BFHs may have totally different results than the current results. In this study, although we excluded the cases reported to be associated with the prevalence of exclusive breastfeeding and/or postpartum depression ^4, 11, 16– 18^ to control confounding factors, we did not perform a further detailed comparison of the women’s characteristics. In addition, although the EPDS has been the most widely used screening tool for postpartum depression in maternity and child services in various countries throughout the world, a EPDS high score does not mean the presence of postpartum depression ^19, 20, 24, 25^.

In conclusion, development of postpartum depression does not seem to be associated with incomplete breastfeeding in Japanese women at our institute. Consequently, there must be other risk factors associated with the development of postpartum depression. A further larger study is needed to clarify these factors.

## Data availability

### Underlying data

Figshare: Breastfeeding and EPDS, https://doi.org/10.6084/m9.figshare.9925070.v1 ^26^

This project contains the following underlying data:

- Dataset 1. Raw data for maternal age, breastfeeding methods and EPDS score recorded from 592 women who hoped to perform exclusive breastfeeding for their babies.

Data are available under the terms of the Creative Commons Zero “No rights reserved” data waiver (CC0 1.0 Public domain dedication).
