# Supplementary material for: Relationship between postpartum depression and lactation status at a Japanese perinatal center: A cross-sectional study
Source: F1000Res. 2020 Jan 30;8:1845. Originally published 2019 Nov 4. [Version 2] doi: 10.12688/f1000research.20704.2 (PMC7059843; doi:10.12688/f1000research.20704.2)
Supplement: Peer review history

# Peer review: Reviewer response for version 2

**DOI:** 10.5256/f1000research.24507.r59356

**Published:** 2020-03-02

**Authors:** Felix Emeka Anyiam

**Affiliations:** 1 University of Port Harcourt , Port Harcourt, Nigeria

**Summary**

 The article titled Relationship between postpartum depression and lactation status at a Japanese perinatal center: A cross-sectional study focused on obstetric records of 809 nulliparous healthy women with vaginal singleton delivery at 37-41 weeks’ gestation between July 2018 and June. The study is relevant, as it set to highlight the benefits of exclusive breastfeeding and its relationship to maternal mental status. Although the present study did not find an association between exclusive breastfeeding and the development of postpartum depression.

**Report**

**A. Title**

 The title for this study is appropriate although I think it should be reversed so that the outcome variable should be at the latter: Relationship between lactation status and postpartum depression at a Japanese perinatal center: A cross-sectional study.

**B. Results**

 it is very difficult to follow on the reading with the results without Tables. The authors should insert result tables for easy reading and comparison.

**C. Discussion**

1. I am not sure what the authors mean by *“* *The current results seemed to be unpredictable for us“ *in the first paragraph.
2. “The influence of synthetic oxytocin on a new mother’s well-being has been also reported previously” should read, “The influence of synthetic oxytocin on a new mother’s well-being **has also been** reported previously.”
3. “The mental status of mothers considered to have low….associate with incomplete breastfeeding seemed to be stable” should read, “The mental status of mothers considered to have low levels of oxytocin **associated** with incomplete breastfeeding seemed to be stable.”
4. “A further larger study is needed to clarify these factors.” Should read, “A further larger study is needed to clarify these **other** factors.

I confirm that I have read this submission and believe that I have an appropriate level of expertise to confirm that it is of an acceptable scientific standard.

# Peer review: Reviewer response for version 2

**DOI:** 10.5256/f1000research.24507.r59333

**Published:** 2020-02-03

**Authors:** Hiroko Iwata

**Affiliations:** 1 Graduate School of Nursing, Chiba University, Chiba, Japan

The author addressed appropriately to the issues raised by the reviewer.

I confirm that I have read this submission and believe that I have an appropriate level of expertise to confirm that it is of an acceptable scientific standard.

# Peer review: Reviewer response for version 1

**DOI:** 10.5256/f1000research.22771.r58361

**Published:** 2020-01-24

**Authors:** Hiroko Iwata

**Affiliations:** 1 Graduate School of Nursing, Chiba University, Chiba, Japan

Methods:

- How did the author control confounding factors? Postpartum depression is reported to have associations with multiple factors, as the author stated in “Introduction”. The author performed univariate analysis (i.e., χ _2_ test and Student’s t-test), not multivariate analysis. If so, results should be interpreted carefully, or some statement as a study limitation is recommended.

Results:

- It is noteworthy and interesting that we could observe a trend of higher score of EPDS and more women with EPDS score of > 9 among exclusive breastfeeding group of women, though the results were non-significant. How did the author interpret those results?

Discussion:

- The first sentence “Although the rate of exclusive breastfeeding may be low in our institute…” does not make sense for readers. The author should contextualize its meaning within the Japanese culture.

I confirm that I have read this submission and believe that I have an appropriate level of expertise to confirm that it is of an acceptable scientific standard, however I have significant reservations, as outlined above.

# Response

**Published:** 2020-01-27

**Authors:** Shunji Suzuki

**Affiliations:** Japanese Red Cross Katsushika Maternity Hospital, Japan

Response to the reviewer,

Many thanks for your careful reading of the manuscript. We appreciate your comments very much. Thank you very much for your suggestions. Based on the queries, we have re-written the many parts of the manuscript.

We have added ‘to control confounding factors’ in the Methods. In addition, we have added a limitation concerning the confounding factors.

We have arranged the first paragraph of the Discussion as suggested.

Thank you very much for considering our paper, again.

Sincerely yours,

Shunji Suzuki, MD

Department of Obstetrics and Gynecology,

Japanese Red Cross Katsushika Maternity Hospital

5-11-12-2 Tateishi, Katsushika-ku, Tokyo 124-0012 Japan

Tel: +81-3-3693-5211

Fax: +81-3-3694-8725

e-mail: czg83542@mopera.ne.jp
